# Emerging clinical importance of the cancer biomarkers kallikrein-related peptidases (KLK) in female and male reproductive organ malignancies

**DOI:** 10.2478/raon-2013-0053

**Published:** 2013-10-08

**Authors:** Manfred Schmitt, Viktor Magdolen, Feng Yang, Marion Kiechle, Jane Bayani, George M. Yousef, Andreas Scorilas, Eleftherios P. Diamandis, Julia Dorn

**Affiliations:** 1 Clinical Research Unit, Department of Obstetrics and Gynecology, Klinikum rechts der Isar, Technische Universität München, Munich, Germany; 2 Ontario Institute for Cancer Research, Transformative Pathology Department, Toronto, Canada; 3 Department of Laboratory Medicine and Pathobiology, University of Toronto, Toronto, Canada; 4 Department of Laboratory Medicine and the Keenan Research Centre in the Li KaShing Knowledge Institute, St Michael’s Hospital, Toronto, Canada; 5 Department of Biochemistry and Molecular Biology, University of Athens; 6 Department of Pathology and Laboratory Medicine, Mount Sinai Hospital, Toronto, Canada

**Keywords:** cancer, proteases, endometrium, ovary, uterus, prostate, testis, cervix, breast

## Abstract

**Background:**

Tumor tissue-associated KLKs (kallikrein-related peptidases) are clinically important biomarkers that may allow prognosis of the cancer disease and/or prediction of response/failure of cancer patients to cancer-directed drugs. Regarding the female/male reproductive tract, remarkably, all of the fifteen KLKs are expressed in the normal prostate, breast, cervix uteri, and the testis, whereas the uterus/endometrium and the ovary are expressing a limited number of KLKs only.

**Conclusions:**

Most of the information regarding elevated expression of KLKs in tumor-affected organs is available for ovarian cancer; depicting them as valuable biomarkers in the cancerous phenotype. In contrast, for breast cancer, a series of KLKs was found to be downregulated. However, in breast cancer, KLK4 is elevated which is also true for ovarian and prostate cancer. In such cases, selective synthetic KLK inhibitors that aim at blocking the proteolytic activities of certain KLKs may serve as future candidate therapeutic drugs to interfere with tumor progression and metastasis.

## Introduction

The human genome encompasses close to 600 different proteases, with about 180 serine proteases (http://degradome.uniovi.es/numbers.html). Serine proteases, *e.g*. plasmin, thrombin, urokinase (uPA), and the KLKs (kallikrein-related serine peptidases) regulate diverse biological processes such as general protein turnover, embryogenesis and pregnancy, blood coagulation, complement activation, and wound healing.^1^ More specifically, serine proteases are involved in cell proliferation and cell signaling, cell migration and invasion, apoptosis and cell death, not only under physiological conditions but also in cancer. Proteases can be released from tissues into the blood, ascitic fluid, alveolar fluid, and cerebrospinal fluid.

KLKs, belonging to the protease clan PA, protease family S1 with subfamily A, located on chromosome 19q13.3–q13.4, are novel, well-suited cancer biomarkers. KLKs are supposed to be of clinical value to identify low-versus high-risk cancer patients, and to predict the course of the cancer disease and response to cancer therapeutics of male and female patients afflicted with reproductive tract malignancies, in addition to cancers of the lung, brain, skin, head and neck, kidney, urinary bladder, and the gastrointestinal tract. In high-risk cancer patient groups, these proteases cannot only be biomarkers for prognosis and therapy response but also act as valuable targets for small size cancer therapeutics, eventually resulting in reduction of the process of tumor cell dissemination and metastasis.^2^

The present, often inefficient, approach to systemic treatment of cancer is commonly referred to as a “trial and error” or “one size fits all” tactic. However, to achieve personalized treatment for cancer patients, one needs meaningful tissue-related or blood-borne biomarkers for the characterization of cancer subgroups, to determine prognosis, response to cancer therapeutics, and to predict severe toxicity related to treatment.^3^ For the cancer biomarkers in focus, a number of the fifteen members of the KLK family are thought to serve as such prognostic and predictive biomarkers, for patients afflicted with various solid malignant tumors.

Highly acknowledged, published evidence supports a strong clinical value of various KLKs to predict the course of certain cancer diseases and in these groups of patients, response to cancer therapy.^1^ Since most of the published data have been collected for ovarian, breast, and prostate cancer, this review focusses on the clinical utility of KLKs for female and male reproductive organ malignancies and the current state-of-the-art regarding incidence of KLKs in afflicted reproductive organs and their potential to predict a patient’s risk to experience untimely disease recurrence, early death, or response/failure to adjuvant or palliative cancer therapy.

In the male urogenital tract, remarkably, all of the KLKs are expressed in the normal prostate and the testes, whereas in females this is only the case for the breast but not for the uterus/endometrium or the ovaries. Most of the information regarding mRNA and/or protein expression of KLKs in tumor-affected organs is available for ovarian cancer; all of the twelve KLKs tested so far were found to be elevated in the malignant state, depicting them as valuable biomarkers to distinguish between the normal and the cancerous phenotype. In contrast, for breast cancer, at the mRNA level, eleven KLKs were found to be down regulated, while *KLK4* and *KLK15* mRNAs were overexpressed compared to normal breast tissue. Interestingly, KLK4 is also overexpressed in cancer of the endometrium, ovary, and the prostate.

In the western world, the incidence of gynecological malignancies is highest for endometrial cancer, followed by cervical and ovarian cancer, while the mortality rate is highest for ovarian cancer, followed by cervical and endometrial cancer.^2^ For cancers of the male reproductive tract encompassing those of the prostate, testis, and penis, prostate cancer is the most frequent cancer in men of older age whereas testicular cancer is most common in younger men.^3^,^4^

KLKs are known to be involved in hormone-dependent cancers of the reproductive system of male or female patients, *e.g*. that of the ovary, breast, prostate.^5^–^8^ Remarkably, in women, the clinical impact of KLK family members as novel biomarkers for screening, diagnosis, prognosis, or therapy response prediction has been mainly studied in ovarian cancer patients.^5^,^9^–^17^

## KLK expression in ovarian cancer

Prognosis of tumors of the ovary is poor, owing to late diagnosis and often inefficient primary debulking surgery of this rare malignancy, but because of rapidly developing chemoresistance as well. In general, the term “ovarian cancer” describes epithelial-surface-type tumors of the ovary, accounting for more than 80% of all solid ovarian tumors. Others, such as sex cord-stromal tumors, germ cell tumors, and metastases from for example gastrointestinal tumors are less common. Two-third of the ovarian cancer patients will develop chemoresistance and disease recurrence within the first 5 years after primary surgery. The therapy of choice is paclitaxel plus carboplatin polychemotherapy. Neither vaginal ultrasonography nor analysis of the tumor-associated antigen CA125 in serum, nor other protein or gene expression analyses of the blood or tumor tissue (*e.g*. ROMA and OVA1) are sufficiently specific to predict the course of the disease or response to systemic adjuvant therapy.^18^–^20^

The stage of ovarian cancer according to the International Federation of Gynecology and Obstetrics (FIGO I–IV) at the time of diagnosis of the disease represents the major traditional prognostic factor. The 5-year survival of early FIGO stage I patients is more than 90%, while survival of patients with FIGO stage III and IV is only 25%. Other important traditional prognostic factors are size of residual tumor mass after cytoreductive surgery histology of the tumor tissue, tumor grade, and presence of ascitic fluid.^21^,^22^ Apart from that, tumor tissue-based biomarkers for screening and risk-group sub classification of early (FIGO I, II) or advanced (FIGO III, IV) ovarian cancer patients reflecting the biology of the tumor are urgently needed.

**FIGURE 1.**
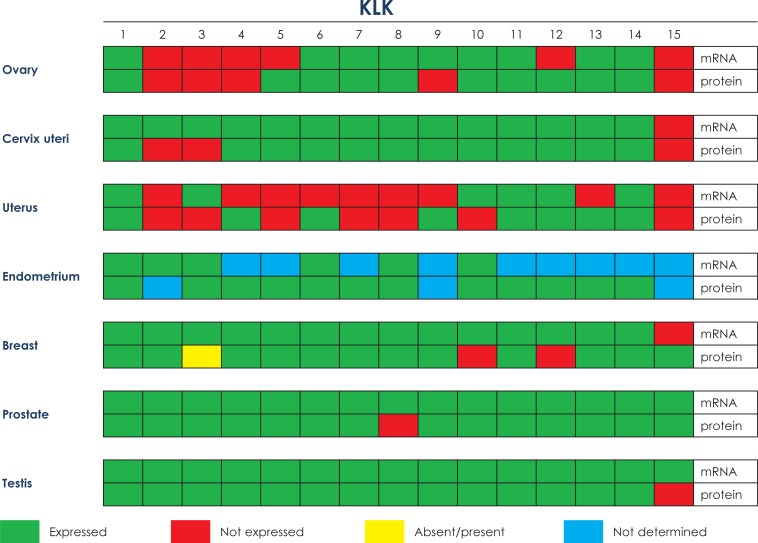
Comparative mRNA and protein expression in normal tissues of the reproductive tract

**FIGURE 2.**
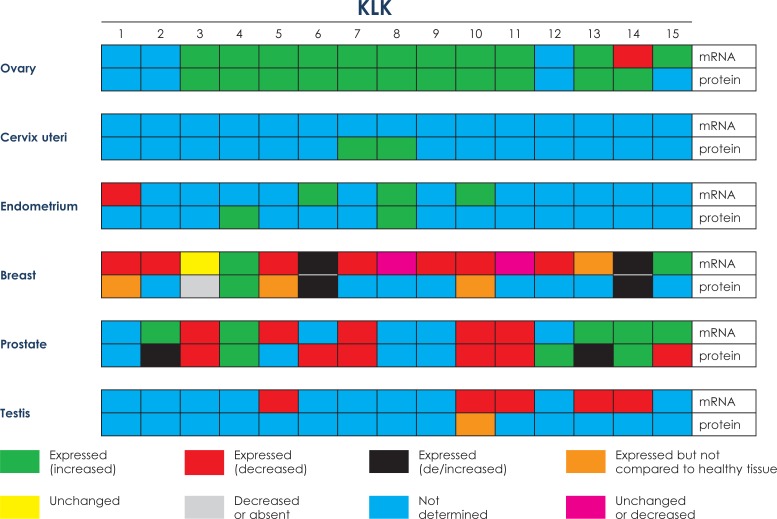
Comparative mRNA and protein expression in cancerous tissues of the reproductive tract

In this respect, in the last decade, mRNA and protein expression of various members of the KLK family has been studied extensively in a variety of normal and diseased human tissues, including the ovary and ovarian cancer 5,9,23 In normal human ovary tissues, *KLK* expression at the mRNA level is highest for *KLK6–8* and *10*, whereas low to moderate expression was noted for *KLK1, 9, 11, 13* and *14* with no expression for *KLK2–5, 12*, and *15.* (Table 1, Figure 1). At the protein level, low to moderate amounts were found for KLK1, 5–8, and 10–14; KLK2–4, 9 and 15 proteins are not expressed (Table 1, Figure 1).^23^ Compared to normal ovarian tissues, concomitant up regulation of twelve (KLK3–11 and 13–15) of the fifteen KLKs at the mRNA and/or protein expression level is characteristic for ovarian cancer (Table 1, Figure 2).^24^–^40^ Regarding the clinical impact of some of the KLKs, expression of KLK4–7, 10 and 15 indicates poor prognosis; KLK8, 9, 11, 13 and 14 are markers of a favorable prognosis. Furthermore, KLK5–8, 10, 11 and 13 are judged as promising predictive ovarian cancer biomarkers.

Seven KLKs (KLK5–8, 10, 11 and 14) are released into the blood, six of these KLKs are also released into peritoneal ascitic fluid (KLK5, 7, 8, 10, 11 and 14) of ovarian cancer patients.41–48 KLK proteins released into the blood or ascitic fluid may also predict the course of early and/or late stage ovarian cancer. KLK8 protein present in blood (serum) indicates a favorable prognosis for the ovarian cancer patient while elevated protein levels of KLK5, 6, 10 and 11 are markers of a poor clinical outcome.^44^,^46^–^49^

## KLK expression in cervical cancer

Owing to well-accepted screening programs and successful therapy of pre-malignant lesions and early stages of cervical cancer, this malignant disease has become a rare disease in the industrialized world, although, malignant tumors of the cervix uteri are still one of the leading causes of death of young women in other countries. Cervical cancer develops stepwise from infection with the human papilloma virus (HPV) and subsequent inefficient immune response to eliminate the virus followed by cervical dysplasia (CIN I–III), subsequently turning into an invasive type of cervical carcinoma.^50^

One of the most important factors to predict the clinical outcome of cervical cancer is clinical stage at the time of diagnosis, thus management of cervical cancer is stage-dependent. Early invasive cervical cancers are subject to surgery, whereby total radical hysterectomy including dissection of the parametries and pelvic lymph nodes, and resection of the vaginal cuff is achieved. In advanced stages of cervical cancer, primary radio-chemotherapy is the therapy of choice^51^, while cancer biomarkers play a lesser role in the management of this cancer disease. Undeniably, no effective prognostic or predictive cancer biomarkers have been established yet for any stage of cervical cancer.^52^

For normal cervix tissue (Table 2, Figure 1), low to moderate mRNA levels were reported for *KLK1–3, 12* and *14*, high ones for *KLK4–11* and *13; KLK15* mRNA is not expressed.^23^,^53^,^54^ Low to moderate KLK protein levels were determined for KLK1 and 4–14; KLK2, 3 and 15 proteins are not expressed. Although KLK mRNA or protein is present in normal cervix tissues, except KLK15, no data have been reported for any KLK mRNA expression in the malignant state (Table 2, Figure 2). Similar, in cervical cancer, no protein expression data were presented for most of the KLKs, except for KLK7 and 8 which are up regulated compared to normal cervix tissue.^55^,^56^ It is worth mentioning that KLK7 protein content increases with the severity of cervical lesions, *i.e*. from cervicitis to low-grade cervical intraepithelial neoplasia, high-grade cervical intraepithelial neoplasia, squamous cervical carcinomas, and even cervical adenocarcinomas.^57^ Obviously, KLK7 could evolve as a useful marker additional to the PAP smear for screening of cervical precursor lesions.^57^

## KLK expression in endometrial cancer

Endometrial cancer, which is a malignancy of the elderly female, derives from the inner glandular layer of the uterus; luckily it is often diagnosed in an early stage of the disease, which leads to expect a favorable clinical outcome. The therapy of choice for endometrial cancer is hysterectomy with bilateral salpingo-oophorectomy, frequently associated with pelvic and paraaortal lymphadenectomy and/or followed by adjuvant radiotherapy. Systemic chemotherapy or endocrine therapy is predominately administered in advanced stages of endometrial cancer, which are rare.^58^

At present, no effective serological or tissue biomarkers do exist to classify endometrial carcinoma patients at risk. Notwithstanding this, immunoenzymometric testing revealed that for eight of the fifteen KLKs low to moderate protein levels were determined in tissue extracts of the uterus (KLK1, 4, 6, 9 and 11–14), seven were not (Figure 1). At the mRNA level, low to moderate values for six of the KLKs were detected (*KLK1, 3, 10–12* and *14*) (Figure 1).

Informative data are available for KLK expression in the normal endometrium, at the mRNA and protein level (Table 3, Figure 1). Six KLK mRNAs (*KLK1–3, 6, 8* and *10)* were found to be expressed, for the other nine KLKs no mRNA expression data have been reported. Assessment by immunohistochemical staining demonstrated protein expression of twelve KLKs (KLK1, 3–8 and 10–14), no data are available regarding protein expression in the normal endometrium of the other three KLKs.^54^ Not much of published information is available regarding the mRNA/protein expression patterns of KLKs in endometrial carcinoma (Table 3, Figure 2). At the mRNA level, *KLK1* was found to be down-regulated whereas *KLK6, 8* and *10* are up-regulated. KLK4 and 8 proteins are up-regulated; no data are available for this malignancy regarding protein expression of the other thirteen KLKs.

## KLK expression in breast cancer

Even though treatment options such as surgery, radiotherapy, chemotherapy/ endocrine therapy, and immunotherapy are currently available, breast cancer remains the second leading cause of cancer-related deaths among women after lung cancer.^59^ Development of breast cancer is a result of multiple genetic changes of epithelial cells and by environmental insults. Several factors may contribute to this malignant transformation process, *e.g*. oncogenes, tumor suppressor genes, hormones, growth factors, and proteases. Serum/plasma-based bio-markers would be helpful for the early diagnosis of breast cancer, for assessment of the course of the disease, prediction of response or resistance to cancer therapeutics, or monitoring of efficacy of therapy.

In fact, several serum-based biomarkers have been described in the literature and are in clinical application, such as CA 15-3, BR 27.29 (CA27.29), carcinoembryonic antigen (CEA), tissue polypeptide antigen, tissue polypeptide specific antigen, or p105HER2 (the shed extracellular domain of HER2).^60^ Although none of these markers is specific or sensitive enough to allow early diagnosis of malignant breast cases or prognosis regarding the clinical course of the breast cancer disease.^61^ Thus, prognostic breast cancer biomarkers in regular clinical practice mainly encompass histomorphological markers (TNM status: tumor size, nodal status, incidence of metastasis, nuclear grading, histological subtype, lymphovascular invasion) plus determination of protein expression of receptors for the steroid hormones estrogen and progesterone but also newer cancer biomarkers such as the multigene panel Oncotype DX and tumor invasion factors uPA/PAI-1.^62^

Extracellular proteases such as uPA, plasmin, matrix metalloproteases, cathepsins, and the KLKs mediate many of the changes in the tumor micro-environment during tumor progression in disrupting the tumor nest-surrounding the basement membrane and the adjacent extracellular matrix (tumor stroma). With the recent discovery of all of the fifteen members of the KLK family, increasing evidence has indicated that KLKs may play pivotal roles in breast cancer progression and metastasis (Table 4, Figure 1,2).^6^,^63^–^65^ In normal breast tissue, all fifteen KLKs have been identified, either at the mRNA and/or the protein level.^23^,^66^*KLK15* mRNA is not expressed by normal breast tissue, low to moderate mRNA levels are detected for *KLK2–5, 9, 12 and 13*, high ones for *KLK1, 6–8, 10, 11 and 14.* No expression of KLK10 and 12 protein was determined but low to moderate levels for KLK1, 4–8 and 13–15 and high levels of KLK9 and 11. Interestingly, KLK3 is not prostate-specific but expressed in a wide variety of other tissues as well, including the breast of about one third of the women.^23^,^67^,^68^ The KLKs are mainly expressed in the breast’s glandular epithelium and some are released into breast secretions, *e.g*. milk of lactating women, breast cyst fluid, and nipple aspirate fluid.^23^,^69^

KLKs are not only involved in breast tissue development but also in various stages of breast cancer development and progression, indicating a regulating role of KLKs in tumor growth and metastasis. In this cancer, most of the KLKs, except KLK4 and KLK15, show reduced mRNA and/or protein expression levels compared to expression of the KLKs in normal breast tissue.^9^,^23^,^66^,^70^,^71^*KLK3, 8* and *11* mRNA expression is not changed in malignant breast tissue compared to normal breast tissue; *KLK1, 2*, and *5–12* mRNA expression is decreased; *KLK4* and *15* are increased. *KLK13* mRNA is expressed in breast cancer tissue but comparison with normal breast tissue has not been made available. For *KLK6* and *14* both increases and decreases in mRNA expression have been reported. At the protein expression level, only KLK4 is elevated compared to normal breast tissue; KLK6 and 14 protein levels were reported either to be lowered or elevated, depending on the study. KLK3 is decreased or absent in breast cancer tumor tissue. Limited data are available for KLK1, 5 and 10 protein expression since expression levels were not compared to expression levels of those proteins in the normal breast tissue. Several other KLKs (KLK2, 7–9, 11–13 and 15) have not been assessed for protein expression in breast cancer tumor tissue yet.

Nine of the fifteen members of the KLK family are considered potential prognostic and/or predictive cancer biomarkers in breast cancer. Five KLKs predict favorable prognosis (KLK3, 9, 12, 13 and 15), four indicate unfavorable, poor prognosis (KLK5, 7, 10 and 14).^5^,^12^,^72^ KLK3 and KLK10 are also predictive markers of response to endocrine therapy.^48^,^73^,^74^ Furthermore, breast cancer risk is associated with presence of single nucleotide polymorphisms (SNP) of KLK2 (Ex5 þ 118C>T) or KLK4 (4207C>G).^75^ No data are available regarding any possible prognostic/predictive value of KLK1, 2, 4, 6, 8 and 11 in breast cancer.

## KLKs in prostate cancer

Following lung cancer, prostate cancer is the second most common cancer and cause of cancer-related deaths in men worldwide.^2^ At time of bioptic diagnosis, tumor stage and Gleason score^76^ plus serum PSA (prostate-specific antigen, also known as kallikrein-related peptidase 3, KLK3) are the most accepted predictors of prognosis of prostate cancer. Treatment strategies may include active surveillance for those cancers that are considered aggressive, surgery with or without a combination of radiation, endocrine therapy or chemotherapy is recommended. Molecular profiling at the genomic, transcriptomic, or proteomic level have identified several potential markers that may distinguish between indolent and aggressive prostate cancers, including *NKX3.1*, *PTEN*, *ETS*, *MYC*, *TP53*, *AR*, *RB1*, and *APC* plus miRNAs as potential prognostic biomarkers.^77^–^82^

In normal prostate tissue, all of the KLKs are expressed at the mRNA level and, except for KLK8, at the protein level as well (Table 5, Figure 1).^23^,^54^ Low to moderate KLK mRNAs levels are found for *KLK4–9, 12* and *13*, high levels for *KLK1–3, 10, 11, 14* and *15*. Low KLK protein expression is reported for KLK4, 5 and 13–15, high ones for KLK1–3, 9 and 11. KLK6, 7, 10 and 12 are expressed as well but expression levels were not scored. In prostate cancer, elevated levels of KLK2, 4 and 13–15 mRNA and/or protein have been reported; KLK3, 5, 7, 10 and 11 are decreased compared to nonmalignant tissue counterparts (Table 5, Figure 2). mRNA expression levels of *KLK 1, 6, 8, 9* and *12* were not determined yet. At the protein level, no information is available for KLK 1, 5, 8 and 9 but for the others with increased levels for KLK4, 12 and 14 versus decreased levels for KLK3, 6, 7, 10, 11 and 15. Conflicting results were reported for KLK2 and 13. Increase of three KLKs (KLK2, 14 and 15) is associated with poor prognosis; KLK4 is a marker of a favorable prognosis. Decreased mRNA or protein levels of KLK2, 3, 5–7, 10, 11, 13 and 15 have been reported of which KLK3 and 15 are markers of a poor prognosis and KLK5 and 11 markers of a favorable prognosis.^70^,^83^,^84^

KLK2 and 3 possess steroid hormone binding sites while KLK1 and 4 possess putative steroid binding elements regulating KLK expression in prostate cancer^85^–^87^; the remaining KLKs do not contain such defined elements.^85^,^86^ DNA-methylation is also involved in KLK regulation as well as non-coding miRNAs.^9^,^88^–^91^

## KLKs in testicular cancer

Testicular cancer, which is affecting men between age 15 and 35 is relatively uncommon in Asia and Africa, but common among Caucasians; the incidence of this cancer increased during the last century for unknown reasons. Testicular cancer is treatable by surgery, radiotherapy, or chemotherapy with a cure rate of ∼95%.^92^,^93^ Even if metastasized to other organs or lymph nodes, the 5-year survival rate is still high (∼72%). For this type of cancer, α-fetoprotein, ß-human chorionic gonadotropin, and lactate dehydrogenase serum markers are useful biomarkers to detect minimal residual disease. Novel biomarkers under investigation, e.g. glypican 3, SALL4, OCT3/4, SOX2, SOX17, OCT3/4, NANOG HMGA1, HMGA2, PATZ1, GPR30, and Aurora B are thought to discriminate between testicular cancer subgroups.^94^–^98^

In the normal testis, all of the fifteen KLKs are expressed at the mRNA level, this is also true for KLK protein expression, except for KLK15 which is not expressed(Table 6, Figure 1).^23^,^54^ Some of the testicular cancer KLK mRNAs have been shown to be of clinical value, such as *KLK5, 10, 11, 13* and *14*, which are all decreased compared to normal tissue expression.^99^–^104^ KLK5 is supposed to be a marker indicating a favorable prognosis.^100^ To date, no study results relating to testicular cancer mRNA expression have been presented for the other ten KLKs; and no results are available relating to the testicular tumor KLK protein levels except for KLK10 (Table 6, Figure 2).

## Future perspectives

KLKs are not only known for their strong biomarker value in prostate, ovarian, breast, and gastrointestinal cancers, regarding prediction of the course of the disease and response to cancer therapy, several KLKs appear to be of clinical value in other malignancies as well, *e.g*. in cancer of the lung, brain, head and neck, the kidney, urinary bladder the endometrium, cervix uteri, and the testes. For several of these malignancies, the tumor tissue-associated KLKs may serve as novel cancer biomarkers in allowing tumor sub classification, diagnosis and prognosis of the cancer disease or prediction of response/failure to cancer-directed drugs. Since, regarding their clinical utility, for most of the KLKs only single reports have been published, validation of KLK gene and protein expression data in independent patient sets on the basis of standard-operating-procedures is a prerequisite before recommendation which of the fifteen KLKs, and for which cancer disease, should be considered for clinical management to support individualized cancer care and treatment. Likewise, in this context, harmonization of methodologies, tools, reagents, and statistics to assess KLK expression in tumors and bodily fluids (plasma/serum, ascitic fluid, lavages) have to be pursued.

At first glance, the KLK peptidases are characterized by high sequence similarities, yet, they show significant differences in their substrate specificities, which will facilitate development of targeted KLK inhibitors. We envision that selective inhibitors to certain KLKs will be developed for future therapeutic application, that aim at blocking their enzymatic activity, in order to interfere with KLK-mediated degradation or activation of other proteins. Nonetheless, one has to bear in mind that KLKs may exist in different enzymatic active and inactive molecular forms. Since reports about the enzymatic state of the various KLKs in different healthy and malignant tissues are scarce at present, the clinical utility of such new synthetic or biological therapeutics is not yet apparent.

## Figures and Tables

**Table:** OVARY, NORMAL

**Expression level (mRNA)**	**KLK number**
Absent	2–5, 12, 15
Low	9, 13
Moderate	1, 11, 14
High	6–8, 10

**Expression level (protein)**	**KLK number**
Absent	2–4, 9, 15
Low	8, 14
Moderate	1, 6, 7, 10, 11
Present	5, 12, 13

**Table:** OVARY, CANCER

**Expression level (mRNA)**	**KLK number**
Not determined	1, 2, 9, 12
Decreased	14
Increased	3–8, 10, 11, 13, 15

**Expression level (protein)**	**KLK number**
Not determined	1, 2, 12
Increased	3–11, 13–15

**Table:** CERVIX UTERI, NORMAL

**Expression level (mRNA)**	**KLK number**
Absent	15
Low	2, 3, 12
Moderate	1, 14
High	4–11, 13

**Expression level (protein)**	**KLK number**
Absent	2, 3, 15
Low	1, 4–8, 10, 13, 14
Moderate	9, 11, 12

**Table:** CERVIX UTERI, CANCER

**Expression level (mRNA)**	**KLK number**
Not determined	1–15

**Expression level (protein)**	**KLK number**
Not determined	1–6, 9–15
Increased	7, 8

**Table:** ENDOMETRIUM, NORMAL

**Expression level (mRNA)**	**KLK number**
Not determined	4, 5, 7, 9, 11–15
Present	1–3, 6, 8, 10

**Expression level (protein)**	**KLK number**
Not determined	2, 9, 15
Present	1, 3–8, 10–14

**Table:** ENDOMETRIUM, CANCER

**Expression level (mRNA)**	**KLK number**
Not determined	2–5, 7, 9, 11–15
Decreased	1
Increased	6, 8, 10

**Expression level (protein)**	**KLK number**
Not determined	1–3, 5–7, 9–15
Increased	4, 8

**Table:** BREAST, NORMAL

**Expression level (mRNA)**	**KLK number**
Absent	15
Low	4, 9, 12
Moderate	2, 3, 5, 13
High	1, 6–8, 10, 11, 14

**Expression level (protein)**	**KLK number**
Absent	3^a^, 10, 12
Low	1, 4, 7, 13
Moderate	2, 5, 6, 8, 14, 15
High	9, 11
Present	3^a^

a Depending on the patient, KLK3 can be expressed or absent.

**Table:** BREAST, CANCER

**Expression level (mRNA)**	**KLK number**
Unchanged	3, 8, 11
Decreased	1, 2, 5–12, 14
Increased	4, 6, 14, 15
Present	13

**Expression level (protein)**	**KLK number**
Not determined	2, 7–9, 11–13, 15
Absent	3
Increased	4, 6, 14
Decreased	3, 6, 14
Present	1, 5, 10

**Table:** PROSTATE, NORMAL

**Expression level (mRNA)**	**KLK number**
Low	5, 6, 9, 13
Moderate	4, 7, 8, 12
High	1–3, 10, 11, 14, 15

**Expression level (protein)**	**KLK number**
Absent	8
Low	4, 5, 13–15
High	1–3, 9, 11
Present	6, 7, 10, 12

**Table:** PROSTATE, CANCER

**Expression level (mRNA)**	**KLK number**
Not determined	1, 6, 8, 9, 12
Decreased	3, 5, 7, 10, 11
Increased	2, 4, 13–15

**Expression level (protein)**	**KLK number**
Not determined	1, 5, 8, 9
Decreased	2, 3, 6, 7, 10, 11, 13, 15
Increased	2, 4, 12–14

**Table:** TESTIS, NORMAL

**Expression level (mRNA)**	**KLK number**
Low	9, 12
Moderate	1–3, 13, 15
High	4–8, 10, 11, 14

**Expression level (protein)**	**KLK number**
Absent	15
Low	1, 2, 4–8, 10, 11, 13
Moderate	3, 9
Present	12, 14

**Table:** TESTIS, CANCER

**Expression level (mRNA)**	**KLK number**
Not determined	1–4, 6–9, 12, 15
Decreased	5, 10, 11, 13, 14

**Expression level (protein)**	**KLK number**
Not determined	1–9, 11–15
Present	10
